# A *Caenorhabditis elegans* assay of seizure-like activity optimised for identifying antiepileptic drugs and their mechanisms of action

**DOI:** 10.1016/j.jneumeth.2018.09.004

**Published:** 2018-11-01

**Authors:** Shi Quan Wong, Alistair Jones, Steven Dodd, Douglas Grimes, Jeff W. Barclay, Anthony G. Marson, Vincent T. Cunliffe, Robert D. Burgoyne, Graeme J. Sills, Alan Morgan

**Affiliations:** a Department of Cellular and Molecular Physiology, Institute of Translational Medicine, University of Liverpool, Liverpool, UK; b Department of Molecular and Clinical Pharmacology, Institute of Translational Medicine, University of Liverpool, Liverpool, UK; c Department of Biomedical Science, University of Sheffield, Sheffield, UK

**Keywords:** *Caenorhabditis elegans*, Calcium channel, Drug screens, Epilepsy, Ethosuximide, GABA receptor, Pentylenetetrazol, Anticonvulsant

## Abstract

**Background:**

Epilepsy affects around 1% of people, but existing antiepileptic drugs (AEDs) only offer symptomatic relief and are ineffective in approximately 30% of patients. Hence, new AEDs are sorely needed. However, a major bottleneck is the low-throughput nature of early-stage AED screens in conventional rodent models. This process could potentially be expedited by using simpler invertebrate systems, such as the nematode *Caenorhabditis elegans.*

**New method:**

Head-bobbing convulsions were previously reported to be inducible by pentylenetetrazol (PTZ) in *C. elegans* with loss-of-function mutations in *unc-49*, which encodes a GABA_A_ receptor. Given that epilepsy-linked mutations in human GABA_A_ receptors are well documented, this could represent a clinically-relevant system for early-stage AED screens. However, the original agar plate-based assay is unsuited to large-scale screening and has not been validated for identifying AEDs. Therefore, we established an alternative streamlined, higher-throughput approach whereby mutants were treated with PTZ and AEDs via liquid-based incubation.

**Results:**

Convulsions induced within minutes of PTZ exposure in *unc-49* mutants were strongly inhibited by the established AED ethosuximide. This protective activity was independent of ethosuximide’s suggested target, the T-type calcium channel, as a null mutation in the worm *cca-1* ortholog did not affect ethosuximide’s anticonvulsant action.

**Comparison with existing method:**

Our streamlined assay is AED-validated, feasible for higher throughput compound screens, and can facilitate insights into AED mechanisms of action.

**Conclusions:**

Based on an epilepsy-associated genetic background, this *C. elegans unc-49* model of seizure-like activity presents an ethical, higher throughput alternative to conventional rodent seizure models for initial AED screens.

## Highlights

• Worms with mutant GABA_A_ receptors exhibit convulsions upon exposure to pentylenetetrazol.
• Convulsions are prevented by the approved anti-epileptic drug, ethosuximide.
• *C. elegans* model is a higher throughput, ethical alternative to rodent seizure models.

## 1 Introduction

Epilepsy is a brain disorder that results in the development of neuronal networks predisposed to the occurrence and recurrence of symptomatic epileptic seizures (Duncan et al., 2006). Epilepsy is one of the most prevalent neurological conditions, with an estimated 50 million sufferers worldwide (WHO, 2018). Although there are currently over 20 approved anti-epileptic drugs (AEDs), these medications provide only seizure relief and are neither disease-modifying nor able to prevent epileptogenesis, the pathobiological process which engenders the development of epilepsy (Brodie et al., 2011; Santulli et al., 2016; Schmidt and Schachter, 2014). Furthermore, although 70% of patients respond to AED treatment, the remaining 30% are refractory to currently available AEDs (Remy and Beck, 2006). Therefore, there is an unmet need for new AEDs that can impact on pharmacoresistant epilepsy and epileptogenesis.

Current AEDs on the market were either discovered through screening in rodent seizure models, or developed as derivatives of pre-existing drugs (Bialer, 2012). Acute seizure induction in mouse and rat models has conventionally been performed either by the subcutaneous administration of the convulsant agent pentylenetetrazol (PTZ) or by trans-corneal electrical stimulation (maximal electroshock, MES) (Löscher, 2011). These procedures have been in use for over 60 years, and despite not recapitulating the complex aetiology of epilepsy, are still used as primary screening platforms to initially assess activity prior to further evaluation in more sophisticated models (Löscher, 2011). However, ethical issues and the financial costs associated with the use of such higher organisms makes them unsuitable for high-throughput early-stage drug screens, especially when large compound libraries such as those employed in modern pharmaceutical development are to be assessed.

These limitations have led to the increasing use of non-mammalian seizure models in recent years (Baraban, 2007; Cunliffe et al., 2015). The fruit fly, *Drosophila melanogaster*, and the zebrafish, *Danio rerio*, have been at the forefront of this area, providing new genetic models of epilepsy and the capacity to perform high-throughput drug screens (Baines et al., 2017; Copmans et al., 2017). In contrast, although the nematode *Caenorhabditis elegans* has been extensively used to model a wide variety of neurological disorders (Chen et al., 2015a), there are relatively few studies where it has been used as a model organism for the study of epilepsy (Jospin et al., 2009; Pandey et al., 2010; Risley et al., 2016; Williams et al., 2004). However, early observations of PTZ-inducible seizure-like phenotypes (Locke et al., 2009; Williams et al., 2004) suggest the possibility of developing *C. elegans* platforms to replace conventional PTZ-induced rodent seizure models for early-stage AED discovery. This presents an attractive alternative, as the use of *C. elegans* offers various important advantages. Specifically, it is predominantly hermaphroditic, has a short generation time (3 days) and lifespan (3 weeks), and is highly tractable genetically. This means that genetic models of epilepsy can be generated much more easily and on a shorter timescale than is achievable with rodent systems (Baraban, 2007; Cunliffe et al., 2015; Takayanagi-Kiya and Jin, 2017). In addition, its facile molecular genetics enables insights into epileptogenic processes and AED mechanisms of action, contributing towards the development of improved AEDs. Lastly, the use of nematode worms is associated with very low costs and the absence of ethical regulations, both of which are factors that limit the utility of rodents.

In view of the attractive prospects of employing *C. elegans* as an alternative platform to overcome the limitations associated with conventional rodent models in AED development, our work seeks to expand on past observations of PTZ-induced seizure-like activity in *C. elegans,* to develop a streamlined nematode worm model suitable for primary AED screening.

## 2 Materials and methods

### 2.1 Materials

All chemicals were obtained from Sigma (Poole, UK), except for DNA polymerase and restriction enzymes, which were from New England Biolabs (Hitchin, UK).

### 2.2 Nematode strains and maintenance

The following *C. elegans* strains were obtained from the *Caenorhabditis* genetics centre (CGC; University of Minnesota, USA): Wild type (WT) Bristol N2, *unc-43 (n498n1186) IV, cca-1 (ad1650) X, unc-25 (e156) III,* and *unc-49 (e407) III*. The *unc-49 (e407)* and *unc-25 (e156) a*lleles are single point mutations that introduce premature stop codons and have been shown to be genetic nulls (Bamber et al., 1999; Jin et al., 1999). The *unc-43 (n498n1186) allele* combines both missense and nonsense mutations and is presumed to be a genetic null (Reiner et al., 1999). The *cca-1 (ad1650)* allele contains a 2.5 kb deletion within the coding region that removes multiple exons and introduces a frameshift and has been shown to be a null mutant (Steger et al., 2005). The double mutant *unc-49*;*cca-1* strain was generated from genetic crosses of *unc-49* (e407) *III* and *cca-1* (ad1650) *X* strains (described below). Worms were grown and maintained on OP50 *E. coli*-seeded nematode growth medium (NGM; 1 mM each of CaCl_2_ and MgSO_4_, 25 mM KH_2_PO_4_, 5 μg/mL cholesterol, and in w/v 2% agar, 0.25% peptone, and 0.3% NaCl) agar in 60-cm plastic petri dishes at 20 °C (Brenner, 1974). All subsequent procedures as described below were performed at 20 °C.

### 2.3 Generation of *unc-49*;*cca-1* double mutants

*C. elegans* males carry a single copy of the X chromosome, hence male *cca-1 (ad1650)* X-linked mutants were derived by mating wild type (WT) Bristol N2 males with self-fertilising homozygous *cca-1 (ad1650)* mutant hermaphrodites. The resultant *cca-1 (ad1650)* males were crossed with *unc-49 (e407)* hermaphrodites to derive *unc-*49;*cca-1* double mutants, selected based on various phenotypic characteristics of the *unc-49* mutation: PTZ sensitivity, reduced thrashes in liquid relative to *cca-1 (ad1650)* mutants, and a “shrinker” phenotype of body contraction and shortening in response to anterior and posterior touch stimuli (McIntire et al., 1993). Homozygous inheritance of the *cca-1 (ad1650)* mutation was verified with PCR primers [forward: 5′−CCGCAATTTGCCCTCCACAT-3′; reverse: 5′- ATGAGGATGGCGAAGAGGACC-3′] which generate differentially-sized products for WT (3310 bp) and mutant (930 bp) alleles. Genotyping of *unc-49 (e407)* homozygosity was performed by initial amplification of the point mutation-harbouring exon with PCR primers [forward: 5′- ATGACCAAGGTTAGGCGACG-3′; reverse: 5′- TCTGGCTACATAACGGCACG-3′], followed by subsequent restriction digestion of products with *MseI*, which distinguishes between WT and mutant *unc-49* alleles based on the number of T^TAA cleavage sites and the size of digested fragments (WT: 259 and 172 bp; mutant: 258, 120, 53 bp).

### 2.4 Age synchronization

Cultured populations on NGM plates wereage-synchronized by bleaching (2 parts 8% commercial alkaline hypochlorite bleach and 1 part 5 M NaOH). Eggs were released from gravid adult worms through lysis by vortexing the mixture every 2 min. for 10 min., followed by pelleting via centrifugation (1 min, 1300 *g*) and subsequent depositing of egg pellet onto seeded NGM plates. The developmental duration from egg to adulthood age day 1 of approximately 3.5 days at 20 °C was adjusted accordingly to attain synchronized populations of older adult worms of defined ages.

### 2.5 Plate-based assay

The induction of head-bobbing convulsions by a plate-based administration of PTZ was adapted from a previously described protocol (Williams et al., 2004). The pre-existing method described the preparation of PTZ-containing NGM agar plates through the direct addition of PTZ to the molten NGM medium to obtain the required working concentrations, which were subsequently seeded with OP50. Modifications herein involved preparation of these plates via spreading of a 50X PTZ stock of the required exposure concentration on the surface of solidified agar plates prior to seeding. For this purpose, PTZ stock solutions were prepared in water at a 50-fold higher concentration than the desired final concentration. Then a volume of this 50X stock equal to 1/50 of the NGM plate volume was added to achieve the desired final 1X concentration. For example, for a 10 ml NGM agar plate one would spread 200 μL of 50 mg/mL PTZ stock to get a 1 mg/mL final PTZ concentration. Unsynchronized young gravid adult nematodes displaying no obvious morphological deterioration were selected visually and transferred to OP50-seeded plates and observed for seizure-like activity for 30 min.

### 2.6 Liquid-based assay

Liquid-based administration of PTZ was performed by incubating individual young (1–3 days old) adult nematodes in 50 μl liquid droplets of PTZ dissolved in Dent’s Ringer solution (DRS; 140 mM NaCl, 1 mM MgCl_2_, 3 mM CaCl_2_, 6 mM KCl, 10 mM HEPES, pH 7.4) containing 0.1% bovine serum albumin (BSA). Droplets were applied to empty plastic petri dishes, with 0.1% BSA included to prevent worms sticking to the plastic. Assays to determine optimal PTZ exposure conditions for convulsion induction utilised PTZ concentrations up to 10 mg/ml for treatment durations up to 60 min. Unless specified otherwise, nematodes were exposed to 7 mg/ml PTZ for 15 min.

### 2.7 Pharmacological treatment

Treatment of nematodes was initiated prior to and continued throughout the duration of PTZ administration to ensure uninterrupted exposure to the pharmacological agent. The validity of the various PTZ-responsive convulsion-prone strains was assayed with the reference AED ethosuximide, which displays anticonvulsive activity in conventional PTZ-induced rat and mouse seizure models (Löscher, 2011). Initial verification of ethosuximide’s anticonvulsive activity was carried out using a previously established longevity-enhancing concentration of 2 mg/ml (Chen et al., 2015b; Evason et al., 2005), following which further optimisation of treatment conditions was performed with up to 4 mg/ml of the drug for treatment durations up to 4 h.

Plate-based administration of ethosuximide was performed by culturing young adult nematodes for 2 days on ethosuximide-containing NGM agar plates prepared by directly diluting a stock solution of the drug dissolved in DRS into molten NGM medium to its required concentration of 2 mg/ml. Liquid-based treatment was carried out by incubating worms in ethosuximide dissolved in DRS/BSA droplets at the specified concentrations and for the specified durations of time. PTZ administration subsequent to either form of pharmacological exposure was performed through a liquid-based approach (see above) in the continued presence of the drug at the same concentration, by transferring nematodes to DRS/BSA droplets containing both PTZ and drug.

### 2.8 Scoring of seizure-like activity

The *unc-49* model exhibits characteristic seizure-like head bobbing movements and a paralysed posterior in response to PTZ (Williams et al., 2004). We defined a single head-bob as an extension and retraction of the anterior pharyngeal region of the worm, along the anterior-posterior axis. These convulsions were stringently scored over 30-second durations in a qualitative and/or quantitative manner where specified. Qualitative scoring assessed the overall presence or absence of convulsions in a worm based on the threshold of three consecutive head bobs, followed by subsequent determination of proportion of the assayed population exhibiting seizure-like activity. The quantitative approach scored the absolute number of head bobs over the same duration. The different scoring approaches complement each other, and concurrent usage strengthens assessment of the phenotype. Phenotypes were scored using a Zeiss Stemi 2000-C microscope (Carl Zeiss Limited, Cambridge, USA) mounted on a Prior OptiScanIITM motorised microscope stage (Prior Scientific Inc, Massachusetts, USA). Worm Tracker (software version 2.0.3.1; http://www.mrc-lmb.cam.ac.uk/wormtracker) was used to record videos of seizure-like activity, which were then analysed manually to generate qualitative and quantitative data.

### 2.9 Thrashing assay

Motility was assessed by quantifying the rate of thrashing in DRS/BSA as previously described (Johnson et al., 2009). A thrash was scored as a head to tail sinusoidal movement, and thrashes were quantified over a 1-min duration following an initial 10-min acclimatisation period in DRS/BSA droplets.

### 2.10 Statistical analysis

All datasets were initially assessed using the Shapiro-Wilk test and were found not to be normally distributed. Hence, the non-parametric Mann-Whitney and Kruskal-Wallis tests were used to determine statistical significance. Analyses were performed on GraphPad Prism version 6 (GraphPad Software Inc.,California, USA). Data is presented as mean values, with standard error of the mean (SEM) where appropriate.

## 3 Results

### 3.1 Development of a liquid-based PTZ assay

Previously, strains harbouring mutations in genes encoding a *C. elegans* GABA_A_ receptor (*unc-49*), glutamic acid decarboxylase (GAD; *unc-25*), and calcium/calmodulin-dependent serine/threonine kinase II (CaMKII; *unc-43*) were observed to exhibit either anterior-type “head bobs” or full body contractions in response to the convulsant PTZ (Williams et al., 2004) (Table 1). To ascertain if we could similarly observe these phenotypes, we replicated the treatment procedure by exposing all three strains to the convulsant agent on PTZ-containing agar plates for 30 min at concentrations ranging from 1 to 10 mg/ml, followed by scoring of the proportion of animals experiencing seizure-like activity, as previously described (Williams et al., 2004). Consistent with these published observations, PTZ-induced convulsions were prominent in at least 20% of each sampled strain over most of the concentrations tested (data not shown). Likewise, we confirmed the previous observation that, unlike these mutant strains, wild type N2 nematodes do not undergo convulsions in response to PTZ exposure.

**Table 1:** Characteristics of PTZ-responsive *C. elegans* mutants used in this study. The human proteins listed are those exhibiting the highest amino acid homology to the corresponding worm protein using BLASTP. The human genes listed are all of those that encode subunits of the functional human receptor/enzyme.

Worm gene	Worm protein	Function	Human protein	Human genes	Human epilepsy mutations	Mutant worm strain	PTZ-induced phenotype
*unc-49*	GABA_A_ receptor	GABAergic inhibitory neuro-transmission	GABA_A_ receptor β1/2/3 subunits	*GABRA1-6* *GABRB1-6* *GABRD GABRE* *GABRG1-3* *GABRP* *GABRQ* *GABRR1-3*	*GABRA1* *GABRB3* *GABRD* *GABRG2*	*unc-49 (e407) III*	Repetitive “head bob” convulsions and paralysed posterior
*unc-25*	Glutamic acid decarbox-ylase (GAD)	GABA synthesis	GAD 1 isoform 1	*GAD1 GAD2*	N/A	*unc-25 (e156) III*	
*unc-43*	Calcium/calmodulin-dependent serine/threonine kinase II (CaMKII)	Phospho-regulation of various physiological processes	CamKII δ subunit isoform δ11	*CAMK2 A/B/D/G*	N/A	*unc-43 (n498 n1186) IV*	Full body convulsions And shrinking

Having successfully replicated the PTZ-induced seizure-like activity reported previously (Williams et al., 2004), we then sought to improve the efficiency and cost-effectiveness of the procedure to facilitate drug screening. Plate-based PTZ administration requires the preparation of PTZ-containing agar plates, which is labour-intensive and incompatible with modern high-throughput drug screens using compound libraries. To overcome these issues, we adapted the treatment procedure from plate-based to liquid-based by incubating the nematodes in small PTZ-containing droplets. This method enables the direct and fresh preparation of PTZ solutions in small volumes, hence not only permitting the use of more stable formulations of PTZ, but also streamlining the procedure by negating the need for additional preparation of drug-containing agar plates.

**Fig. 1:**
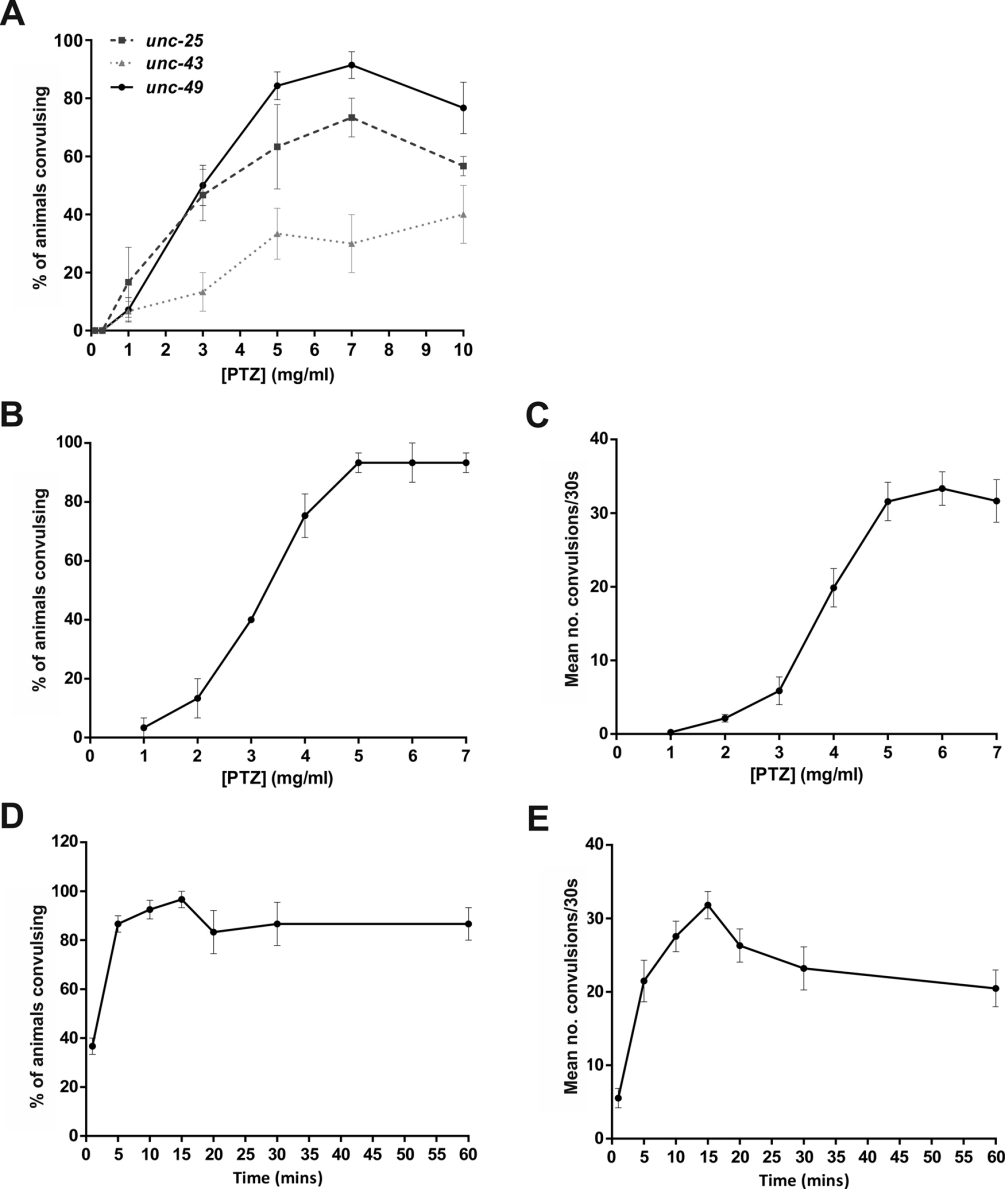
Optimization of the liquid-based assay. (A) Worms were exposed to the indicated concentrations of PTZ in solution for 15 min and then the proportion of worms experiencing head-bobbing convulsions (*unc-25*, *unc-49*) or full body convulsions (*unc-43*) in 30 s was scored. *unc-49* mutants exhibited the highest incidence of seizure-like activity and were selected for further optimization. (B and C) *unc-49* mutants were exposed to the indicated concentrations of PTZ for 15 min and then both the proportion of worms exhibiting head-bobbing convulsions (B) and the number of convulsions experienced per animal (C) was measured over 30 s. (D and E) *unc-49* mutants were exposed to 7 mg/ml PTZ for the indicated times and then both the proportion of worms exhibiting head-bobbing convulsions (D) and the number of convulsions experienced per animal (E) was measured over 30 s. Data shown were pooled from three independent experiments (*n* = 27–30 worms in total per concentration or time point).

An initial 5-point PTZ concentration-response analysis was performed on all three mutant strains using the liquid droplet method, which showed a concentration-dependent induction of convulsions that peaked around 7 mg/ml (Fig. 1A). Example movies of the head-bobbing convulsions exhibited by *unc-49* mutants and the full body convulsions exhibited by *unc-43* mutants are shown in Supplementary videos S1 and S2, respectively. Wild type N2 worms did not undergo convulsions at any of the tested PTZ concentrations (data not shown). As *unc-49* mutants exhibited the highest incidence of seizure-like activity (Fig. 1A), and as *unc-49* is homologous to human GABA_A_ receptor subunits that are known to be mutated in various forms of epilepsy (Table 1), we chose this strain for further optimisation. A more detailed, 7-point PTZ concentration-response experiment was then performed, confirming that the incidence of convulsions was maximal at 5–7 mg/ml PTZ (Fig. 1B). Up to this point, seizure-like activity was solely scored as the proportion of animals exhibiting convulsions, as previously described (Williams et al., 2004), so we introduced a new approach to improve stringency by concurrently quantifying the number of head bobs exhibited by each animal over the 30-second scoring duration. In keeping with the original scoring method, the frequency of PTZ-induced convulsions was concentration-dependent, culminating and stabilising at 5–7 mg/ml (Fig. 1C). We subsequently selected 7 mg/ml based on the rationale that seizure-like activity would be most robustly induced at the highest convulsant concentration within this range. To determine whether the 15-minute exposure to PTZ used in these initial experiments was optimal, a time course experiment was performed. Both the highest proportion of animals experiencing convulsions and the highest number of convulsions per animal were observed after 15 min of PTZ exposure (Fig. 1D, E). After longer periods of PTZ exposure, the number of convulsions per animal began to decline, due to progressive paralysis. Therefore, exposure of *unc-49* mutants to 7 mg/ml PTZ for 15 min was established as the standard condition to be used for the liquid-based assay

**Fig. 2:**
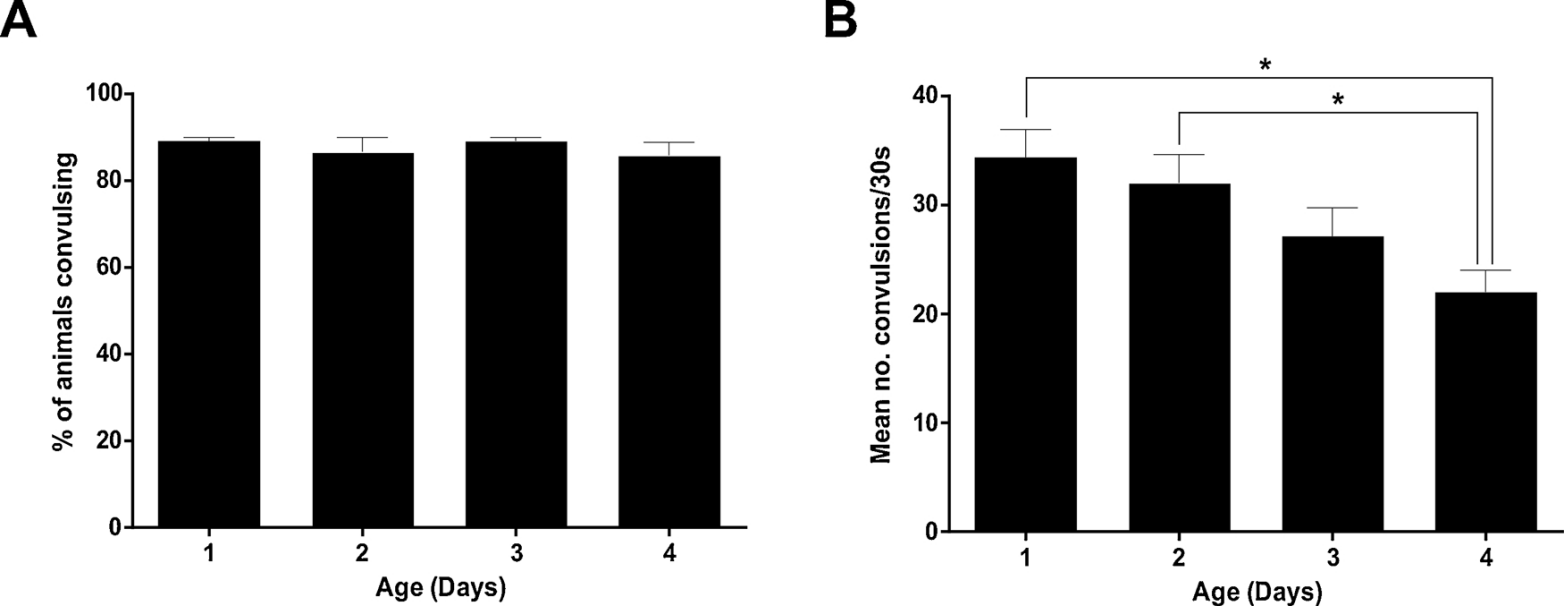
Responses to PTZ are not age-dependent in younger worms. *unc-49* mutants were age-synchronised by bleaching and grown to the indicated ages before being exposed to 7 mg/ml PTZ in solution for 15 min. At this point, the proportion of worms exhibiting head-bobbing convulsions (A) and the number of convulsions experienced per animal (B) were measured over 30 s. No age-associated difference in PTZ responsiveness was seen when the proportions of animals exhibiting convulsions were scored (A); but the mean number of convulsions per worm (B) was significantly reduced in 4-day-old adults as compared to younger ages of days 1 and 2. Data shown were pooled from three independent experiments (n = 27–30 worms in total per adult age point). Statistical analysis was performed with the Kruskal-Wallis test with Dunn's correction for multiple comparisons; *, p < 0.05.

Next, we sought to establish if there is an age-dependent variability in PTZ sensitivity in order to ascertain firstly if age-synchronized populations are required, and if so, the stage of the worm’s lifespan that responds most robustly to PTZ. The optimised conditions described above (Fig. 1) were determined using unsynchronized young adult nematodes (approximately 1–3 days old), which were selected based on the absence of visual morphological deterioration. Age-response analysis was therefore performed with age-synchronized populations mirroring these approximate ages. Although no significant age-related differences were detectable based on scoring the percentage of animals exhibiting convulsions in response to PTZ (Fig. 2A); there was a significant decline in the number of convulsions exhibited per animal in 4-day-old worms (Fig. 2B). This indicates that young adult nematodes of mixed ages between days 1–3 may be utilised with negligible effect on PTZ-mediated induction of seizure-like activity, thereby eliminating the need for laborious age-synchronization methods to be performed in advance.

### 3.2 Optimization of the liquid-based assay for AED screening

**Fig. 3:**
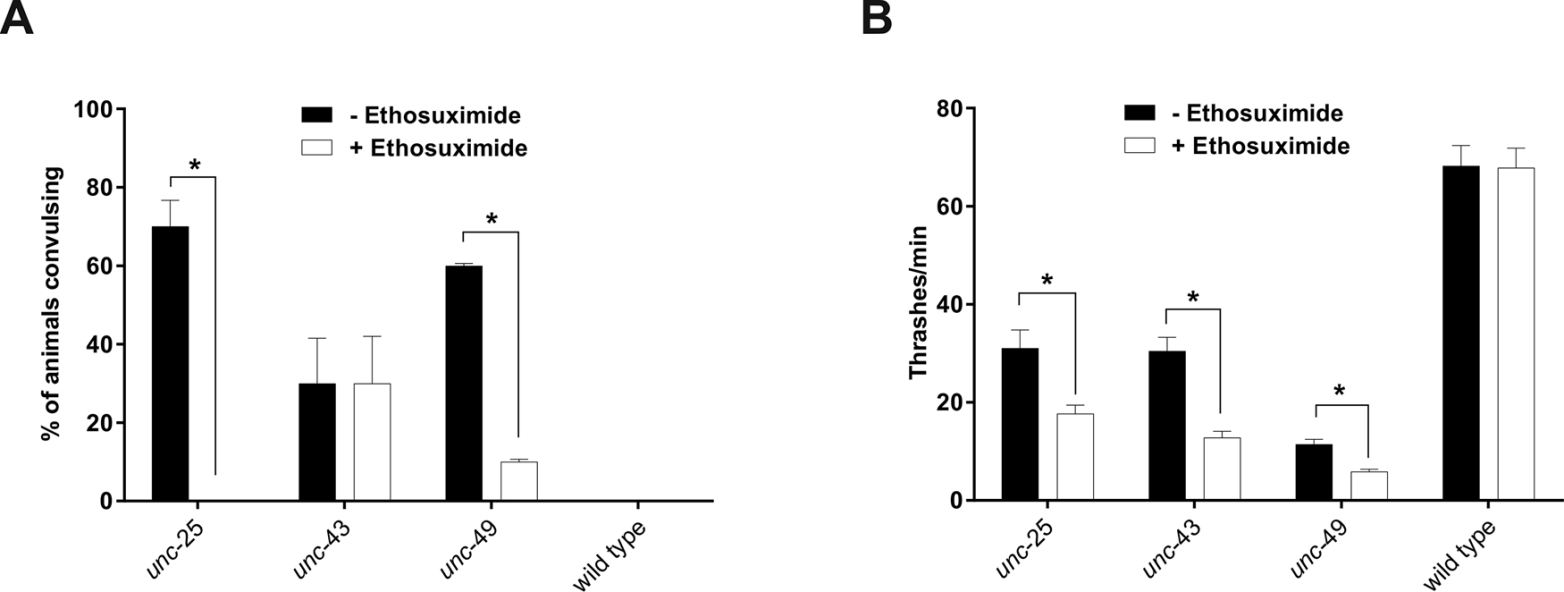
Ethosuximide reduces seizure-like activity in GABAergic mutant strains. (A) Worms were cultured on agar plates containing ethosuximide or vehicle control for 2 days before incubation in a 7 mg/ml PTZ solution for 15 min. The proportion of worms experiencing head-bobbing convulsions (*unc-25*, *unc-49*) or full body convulsions (*unc-43*) in 30 s was then scored. The incidence of PTZ-induced seizure-like activity was significantly reduced by ethosuximide treatment in *unc-25* and *unc-49* mutants, but not *unc-43* mutants. Wild type N2 worms did not undergo convulsions in response to PTZ. (B) Worms were cultured on agar plates containing ethosuximide or vehicle control for 2 days before incubation in Dent’s Ringer solution for 10 min. The number of thrashing movements made by each worm in 60 s was then scored. Ethosuximide significantly reduced the thrashing frequency of all mutant strains, but had no effect on wild type N2 worms. Data shown were pooled from three independent experiments (*n* = 30 worms in total per strain per condition), with statistical comparisons made via the Mann-Whitney test; *, p < 0.05.

Next, the ability of PTZ-induced seizure-like activity to be suppressed by a known anticonvulsant was evaluated, in order to validate the worm model as a potential AED screening platform. The AED ethosuximide was chosen, as its anticonvulsive activity against PTZ-induced seizures in rodent models is long established (Löscher, 2011). As this was the first attempt to demonstrate anticonvulsive activity of ethosuximide in *C. elegans*, we selected 2 mg/ml as an initial concentration, based on previous reports of lifespan extension in nematodes at ethosuximide concentrations between 1 and 4 mg/ml (Chen et al., 2015b; Choi et al., 2013; Collins et al., 2008; Evason et al., 2005; Tauffenberger et al., 2013). These longevity effects had been observed with animals grown continuously on ethosuximide-containing agar plates, so we initially adopted a similar long-term treatment procedure. Unsynchronised, mixed stage mutant worms were cultured on NGM agar plates containing 2 mg/ml ethosuximide for two days prior to incubation in ethosuximide- and PTZ-containing liquid droplets, to ensure continuous exposure to the AED. To distinguish toxic from therapeutic effects of ethosuximide, we applied visual and phenotypic assessments at the end of AED pre-treatment and/or PTZ exposure. Toxicity was determined by visual observations of abnormal stiff movement or immobility, and a weak response to touch. Conversely, a therapeutic effect was defined by a reduction in or abolition of convulsions in the presence of movement and a normal response to touch. The absence of convulsions in immobile worms was only considered therapeutic if a robust response to touch was observed, indicative of non-toxicity. In line with these criteria, ethosuximide exerted a significant therapeutic reduction in the incidence of PTZ-induced seizure-like activity in both *unc-25* and *unc-49* mutants, but had no discernible effect on *unc-43* worms (Fig. 3A).

Drowsiness and sedation are reported adverse effects associated with ethosuximide treatment in patients (Patsalos, 2013). Therefore, we investigated if the anticonvulsive effects of the drug observed in *unc-25* and *unc-49* strains may be attributed to mutant-specific impairment of movement that does not occur in *unc-43* worms. Locomotion was therefore assessed via the frequency of thrashes in a liquid medium in order to test this possibility. Example movies showing the thrashing activity of N2 and *unc-49* strains are shown in Supplementary video S3 and S4, respectively. Ethosuximide treatment had no significant effect on wild type N2 worms but reduced the thrashing rates of all three *unc* mutant strains by approximately 50% (Fig. 3B). However, as ethosuximide decreased *unc-43* motility despite having no discernible anticonvulsive activity in this mutant, it is unlikely that the protection from seizure-like activity in *unc-25* and *unc-49* mutants is simply due to impaired movement.

**Fig. 4:**
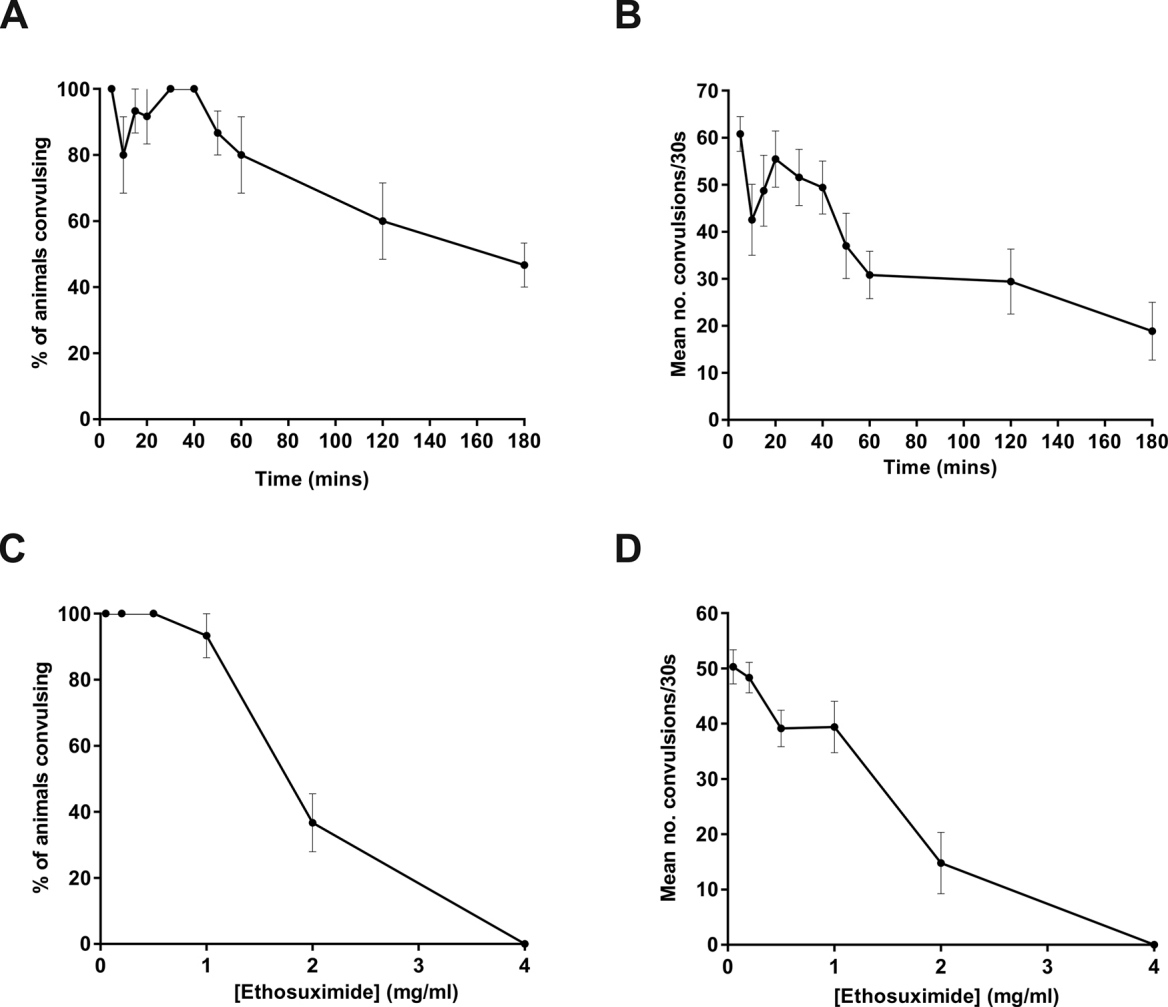
Optimization of liquid-based AED screening conditions using ethosuximide in *unc-49* mutants. (A and B) *unc-49* mutants were pre-incubated in a solution containing 2 mg/ml ethosuximide for the indicated times; or (C and D) were pre-incubated for 2 h in the indicated concentrations of ethosuximide. The worms were then exposed to 7 mg/ml PTZ for 15 min and the proportion of worms exhibiting head-bobbing convulsions (A and C) and the number of convulsions experienced per animal (B and D) were measured over 30 s. Complete abolition of convulsions was achieved with a 2-hour incubation at 4 mg/ml ethosuximide. Data shown were pooled from three independent experiments (*n* = 10–15 worms in total per drug treatment time-point or concentration).

Ethosuximide administration via a two-day agar plate-based exposure (Fig. 3) is time-consuming and incompatible with modern compound library screening approaches. In order to develop a faster, liquid-based method, we performed a time-course investigation by pre-incubating *unc-49* mutants in ethosuximide-containing droplets for up to 3 h prior to PTZ exposure. An initial concentration of 2 mg/ml ethosuximide was chosen, as this was effective in the agar plate-based experiments (Fig. 3). However, given that the optimal anticonvulsive concentration of ethosuximide when applied in solution was yet to be determined, variability was anticipated in responses to the drug at various time-points. Despite this, a time-dependent reduction in both convulsion incidence and absolute convulsion number was observed after 30 min of drug treatment, although seizure-like activity was not completely abolished even after the longest treatment duration of 3 h (Fig. 4A and B). As the frequencies of head bobbing convulsions appeared to stabilise after an hour of drug treatment (Fig. 4B), we selected an intermediate duration of 2 h as the preferred drug exposure time. This 2-hour pre-treatment protocol was then used to determine the optimal anticonvulsant concentration of ethosuximide, demonstrating a concentration-dependent reduction in convulsions that reached complete abolition at 4 mg/ml (Fig. 4C and D). Importantly, this convulsion-free state of *unc-49* worms was not due to ethosuximide-induced paralysis, as the movement of the worms in PTZ solution actually became more coordinated after ethosuximide treatment (Supplementary video S5).

**Fig. 5:**
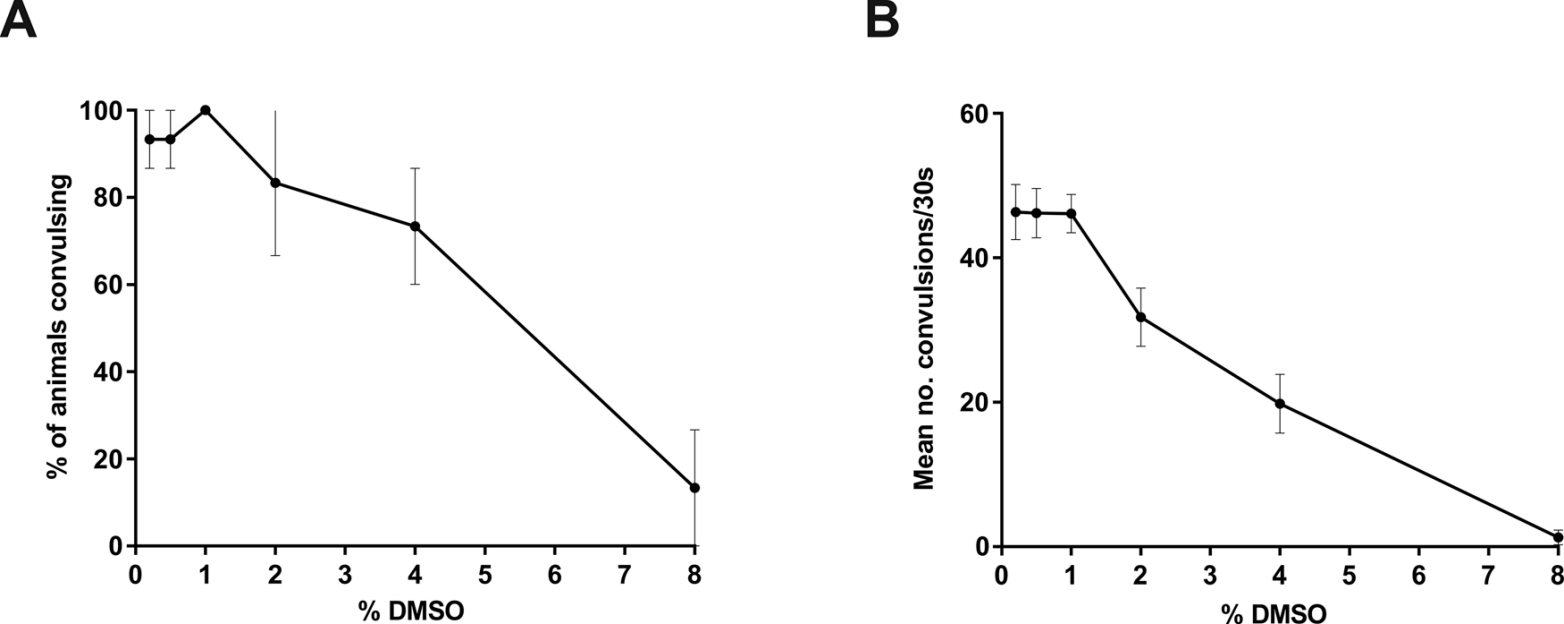
Identification of non-toxic DMSO levels for drug screens in *unc-49* mutants. Worms were exposed to the indicated concentrations of DMSO throughout a 2-hour pre-incubation period and a 15-minute PTZ exposure period. (A) The proportion of worms exhibiting convulsions and (B) the mean number of convulsions per animal were then scored over 30 s. PTZ responsiveness was unaffected up to 1% DMSO, but higher DMSO concentrations resulted in toxicity that reduced sensitivity to PTZ. Data shown were pooled from three independent experiments (n = 10–15 worms in total per DMSO concentration).

Ethosuximide is water-soluble and was therefore solubilised in an aqueous medium to optimise these drug screening conditions. However, chemicals from compound libraries are mostly maintained and screened as solutions solubilised with dimethyl sulfoxide (DMSO) as a vehicle, necessitating the *unc-49* platform to be further adapted for a broader screening applicability. Given that DMSO can have toxic effects on *C. elegans* (Boyd et al., 2010), it was important to determine the tolerable threshold of DMSO that does not cause observable toxicity according to the aforementioned criteria, and does not affect responses to PTZ. Therefore, screening conditions were mimicked by exposing *unc-49* mutants to varying concentrations of DMSO throughout the intended 2-hour drug treatment and 15-minute PTZ exposure windows. Convulsions induced by PTZ were unaffected by up to 1% DMSO, but further increases in DMSO resulted in a concentration-dependent reduction in response to the convulsant (Fig. 5). This decreased responsiveness to PTZ was likely due to DMSO toxicity, as these higher concentrations of DMSO resulted in severe movement defects and visible toxicity-associated behavioural abnormalities as defined previously. Furthermore, these phenotypic aberrations were clearly distinguishable from the therapeutic-associated more coordinated movement observed in ethosuximide-treated worms. Since sensitivity to PTZ was evidently impaired with ≥ 2% DMSO, future AED screens using DMSO-solubilized compounds should ideally be performed using no more than 1% DMSO.

### 3.3 Chemical genetic testing of AED mechanism of action

**Fig. 6:**
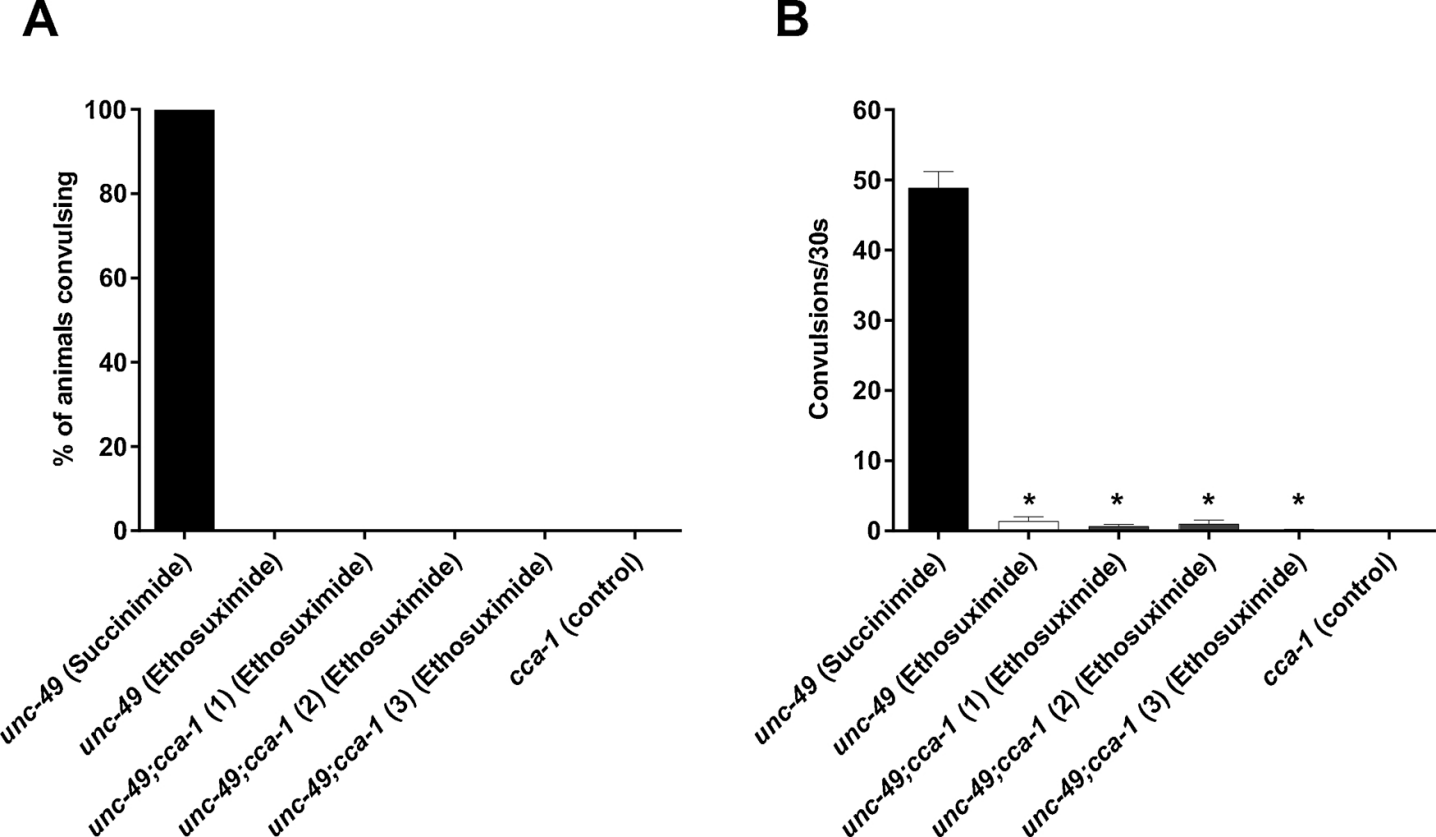
Ethosuximide does not mediate anticonvulsant effects through the T-type calcium channel, CCA-1. Three *unc-49* lines harbouring a loss-of-function mutation in the T-type voltage-gated calcium channel-encoding *cca-1* gene (*unc-49*;*cca-1*) were generated. These and the parent *unc-49* single mutant strain were pre-incubated for 2 h in a solution containing either 4 mg/ml ethosuximide or 4 mg/ml succinimide, before exposure to PTZ for 15 min. The loss of these channels did not affect protection from seizure-like activity by ethosuximide, as the drug (A) prevented overall convulsions and (B) significantly reduced the convulsion rates in all lines when compared to *unc-49* worms treated with the inert succinimide control (p < 0.05). Furthermore, anticonvulsant activity in all lines were comparable to *cca-1*-intact ethosuximide-treated *unc-49* controls (p > 0.05). Data shown were pooled from three independent experiments, with convulsion rates compared via the Kruskal-Wallis test with Dunn’s multiple comparison correction (*n* = 13–15 worms in total per strain per condition); *, p < 0.05.

One major advantage of using a *C. elegans* model is the ability to use powerful genetic approaches to examine potential mechanisms of drug action *in vivo*. This possibility was therefore assessed with the new assay. Ethosuximide has been prescribed for 60 years and is still used as a first-line treatment against absence epilepsy in children, but its molecular mechanism of action remains unclear. Although ethosuximide has been suggested to exert its antiepileptic effect by inhibiting T-type voltage-gated calcium channels (Coulter et al., 1989a,b 1990; Gomora et al., 2001; Lacinova et al., 2000), this is controversial and several alternative targets have been suggested (Crunelli and Leresche, 2002; Fohlmeister et al., 1984). To investigate if these channels constitute the AED’s molecular target against PTZ-induced convulsions in *C. elegans*, we deleted the worm T-type calcium channel-encoding *cca-1* orthologue in *unc-49* mutants by genetically crossing them with a loss-of-function *cca-1* mutant to generate double mutant *unc-49*;*cca-1* animals. Three independently-derived lines were genetically verified for both mutations (Supplementary Figure S1), and assayed to validate the reproducibility of responses to drug treatment and PTZ exposure. The single *cca-1* mutant did not exhibit seizures in response to PTZ, similar to wild type strains (Fig. 6; Supplementary video S6). Loss of functional CCA-1 had no significant effect on the ability of PTZ to evoke convulsions in double *unc-49*;*cca-1* mutants (Supplementary video S7). The anticonvulsant activity of 4 mg/ml ethosuximide was unaffected by mutation of *cca-1*, as both the proportion of convulsing worms (Fig. 6A) and the mean number of head bobs exhibited per animal (Fig. 6B) were comparable in double *unc-49*;*cca-1* mutants and single *unc-49* mutants containing wild type CCA-1. Importantly, the same concentration of the chemically similar but biologically inert molecule succinimide conferred no protection from PTZ-induced convulsions in single *unc-49* mutants (Fig. 6) or double *unc-49*;*cca-1* mutants (Supplementary video S7), demonstrating that the anticonvulsant activity of ethosuximide was specific to the drug and not due to non-specific effects of the treatment conditions. We therefore conclude that ethosuximide does not prevent PTZ-induced convulsions in *unc-49* mutants by inhibiting T-type calcium channels.

## 4 Discussion

Building on previously-reported observations of PTZ-induced seizure-like activity in GABAergic and CaMKII *C. elegans* mutants (Williams et al., 2004), we have generated a refined platform using *unc-49* GABA_A_ receptor mutants. Using our streamlined approach, characteristic head-bobbing convulsions were initiated through liquid-based PTZ exposure, resulting in a two-fold reduction in the exposure time required for optimal induction of seizure-like activity compared to the agar plate-based method used in the original study. Our liquid-based method of administering both PTZ and AED through droplet incubation offers two further advantages. Firstly, throughput is increased and cost is reduced, since tedious and more expensive PTZ and drug plate preparations are not required. Secondly, our refined technique is better suited to the use of compound libraries. Since the latter are typically prepared as stock solutions in DMSO in microtitre plates, diluted solutions for screening can be easily and directly prepared in micro-titre formats, especially with the use of robotic platforms (Buckingham et al., 2014). Furthermore, we have demonstrated that PTZ responsiveness is unaffected by DMSO concentrations up to 1%, thereby facilitating screening of DMSO-solubilised compound libraries. Additional streamlining of the screening process could potentially be achieved using robotic systems for automated culturing and handling of nematodes, which are also adaptable for micro-titre plate configurations (Bazopoulou et al., 2017; Boyd et al., 2010; Xian et al., 2013). Although we have demonstrated that precise age synchronization is not essential for our platform, since 1- to 3-day-old adult worms respond similarly to PTZ, current technologies enabling the sorting of *C. elegans* by age and/or size could potentially be used to further enhance the assay by facilitating automated selection of young adult nematodes (Boyd et al., 2010; Pulak, 2006; Rezai et al., 2012).

Despite these advantages, one caveat of our system is the use of visual scoring of the characteristic head-bobbing convulsions. Manual scoring of convulsions is inherently liable to subjectivity, and so the implementation of an automated scoring method would standardize scoring parameters and greatly improve the throughput of our screening platform. Given that such automated scoring technologies have already been developed for other phenotypes in *C. elegans* (Buckingham et al., 2014), this is likely achievable for the head-bobbing phenotype with the implementation of algorithms and parameters to strictly differentiate between seizure-like activity and other behaviours. A further potential limitation of our system is the use of the *unc-49* GABA_A_ receptor mutant background, which may produce false negatives for AEDs that directly or indirectly target GABA_A_ receptors. This issue is mitigated to some extent by the fact that *unc-49* is only one of seven GABA_A_ receptor-encoding genes in the worm genome (others being *gab-1*, lgc-35, *lgc-36, lgc-37, lgc-38* and *exp-1*), so GABA_A_-receptor-binding drugs should still be able to exert effects (Gendrel et al., 2016). Finally, some classes of AEDs may have no activity in our assay because their target proteins are not expressed in worms. For example, the *C. elegans* genome does not encode voltage-gated sodium channels (Bargmann, 1998), which are the most common molecular target of currently prescribed AEDs. However, if the goal is to discover new AEDs with novel molecular targets, perhaps using an animal model that lacks one of the best established drug targets could be argued to be an advantage.

In order for a worm assay to be potentially useful for novel AED screening, clinically approved AEDs must first be shown to protect against convulsions as a proof of principle. Here, we validated the *unc-49* platform by replicating the long-established anticonvulsant effect of ethosuximide seen in traditional rodent PTZ seizure models (Chen et al., 1963; Löscher, 2011). Pre-incubation for 2 h in a bathing solution of 4 mg/ml ethosuximide greatly reduced seizure-like activity and also improved the uncoordinated movement of PTZ-treated *unc-49* mutant worms. Based on previous studies of ethosuximide accumulation in *C. elegans* (Evason et al., 2005) and the linear relationship between ethosuximide dosage and serum/CSF concentration in humans (Patsalos, 2005), we estimate that the internal concentration of ethosuximide in worms bathed in a 4 mg/ml solution is approximately 440 μM. This value is within the known human therapeutic serum concentration range of 280–700 μM (Goren and Onat, 2007), further supporting the pharmacological validity of our worm assay. Chemical screens have previously been performed in both heat- and electroshock-induced *C. elegans* models of seizure-like activity (Pandey et al., 2010; Risley et al., 2016). However, the anticonvulsive effects of known AEDs against heat-induced convulsions was not demonstrated. Although several established AEDs were assessed in the electroshock model, protective effects were evaluated based on improvement of the recovery time from convulsions after removal of the electric stimulus, as opposed to inhibition of convulsions *per se*. Despite these limitations, both models have potential utility for compound library screening. Our system, using PTZ exposure in *unc-49* mutants, offers a complementary platform that is already validated using a currently prescribed AED based on a direct readout of convulsion reduction. Applications of our model include unbiased compound screens to find novel anticonvulsant molecules as well as chemical modification of existing AEDs to facilitate drug repurposing. Indeed, we have recently used the *unc-49* assay to rapidly pre-screen ethosuximide-based compounds for bioactivity before subsequent testing for increased neuroprotective potency (Wong et al., 2018). In view of the large number of rodents used as acute seizure models in AED discovery, *C. elegans* assays could be a more economical and ethical alternative.

The ease of genetic manipulation in *C. elegans* and the availability of mutants covering most of the genome facilitates the rapid creation of genetic epilepsy models. Indeed, the *unc-49* mutant characterised herein (Bamber et al., 1999) and the aforementioned *acr-2* mutant (Jospin et al., 2009) contain mutations in *C. elegans* homologues of human GABA_A_ and nicotinic acetylcholine receptor genes, respectively, which have been linked to various forms of epilepsies (Audenaert et al., 2006; Baulac et al., 2001; Chen et al., 2009; Cossette et al., 2002; Epi et al., 2013; Fusco et al., 2000; Gambardella et al., 2003; Harkin et al., 2002; Hirose et al., 1999; Kananura et al., 2002; Lachance‐Touchette et al., 2011; Leniger et al., 2003; Lerche et al., 2013; Marini et al., 2003; Phillips et al., 2001; Rozycka et al., 2003; Sáenz et al., 1999; Steinlein et al., 1997, 1995; Sun et al., 2008; Wallace et al., 2001; Wang et al., 2017). These links to genetic epilepsies therefore provide a basis for the clinical relevance of both *acr-2* and *unc-49* platforms. Furthermore, chemical genetic approaches can be performed rapidly in *C. elegans* to shed light on the mechanisms of action of currently prescribed AEDs, which are often poorly understood. Indeed, ethosuximide has been variously suggested to inhibit T-type calcium channels (Coulter et al., 1989a, 1990, 1989b; Gomora et al., 2001; Lacinova et al., 2000), voltage-gated sodium channels and potassium channels (Fohlmeister et al., 1984); to activate forkhead box O (FOXO) transcription factors (Chen et al., 2015b); and to modulate the phosphoinositide 3-kinase/AKT/Wnt/β-catenin signalling pathway (Tiwari et al., 2015). Our proof-of-concept chemical genetics investigation has clearly demonstrated that ethosuximide’s anticonvulsant effect is not mediated by the *C. elegans* T-type calcium channel CCA-1. The inability of ethosuximide to rescue the full-body convulsions in *unc-43* null mutants could potentially suggest that UNC-43 (CAMKII) or a CAMKII-phosphorylated substrate protein(s) may be the molecular target of ethosuximide. However, there are no published data linking ethosuximide to CAMKII, so evidence for this proposition is lacking. Nevertheless, although we have not yet identified ethosuximide’s molecular target, the ease of conducting genome-wide genetic screens in *C. elegans* suggests that this could be feasibly achieved using our assay.

## 5 Conclusions

In conclusion, we have developed a streamlined assay in *C. elegans* suitable for early-stage AED screening. We envisage that this could be used in future as the first pass in a pipeline for drug discovery, with promising compounds being prioritised for validation in conventional rodent models, thus reducing the ethical and financial costs of AED screening. In addition, the *C. elegans* system enables rapid identification of the mechanisms of action of such validated novel AEDs via chemical genetics.

## Competing financial interests

The authors declare no competing interests.

## Author contributions

SW, AJ, SD and DG performed the experiments. SW, AJ, AD, JWB, RDB, AGM, VTC GJS and AM analysed and interpreted the data. AM and GJS conceived and designed the experiments. SW and AM wrote the manuscript with input from all of the other authors.
