# Supplementary material for: A Caenorhabditis elegans assay of seizure-like activity optimised for identifying antiepileptic drugs and their mechanisms of action
Source: J Neurosci Methods. 2018 Nov 1;309:132–42. doi: 10.1016/j.jneumeth.2018.09.004 (PMC6200019; doi:10.1016/j.jneumeth.2018.09.004)
Supplement: Supplementary file 1 [file mmc1.pdf]

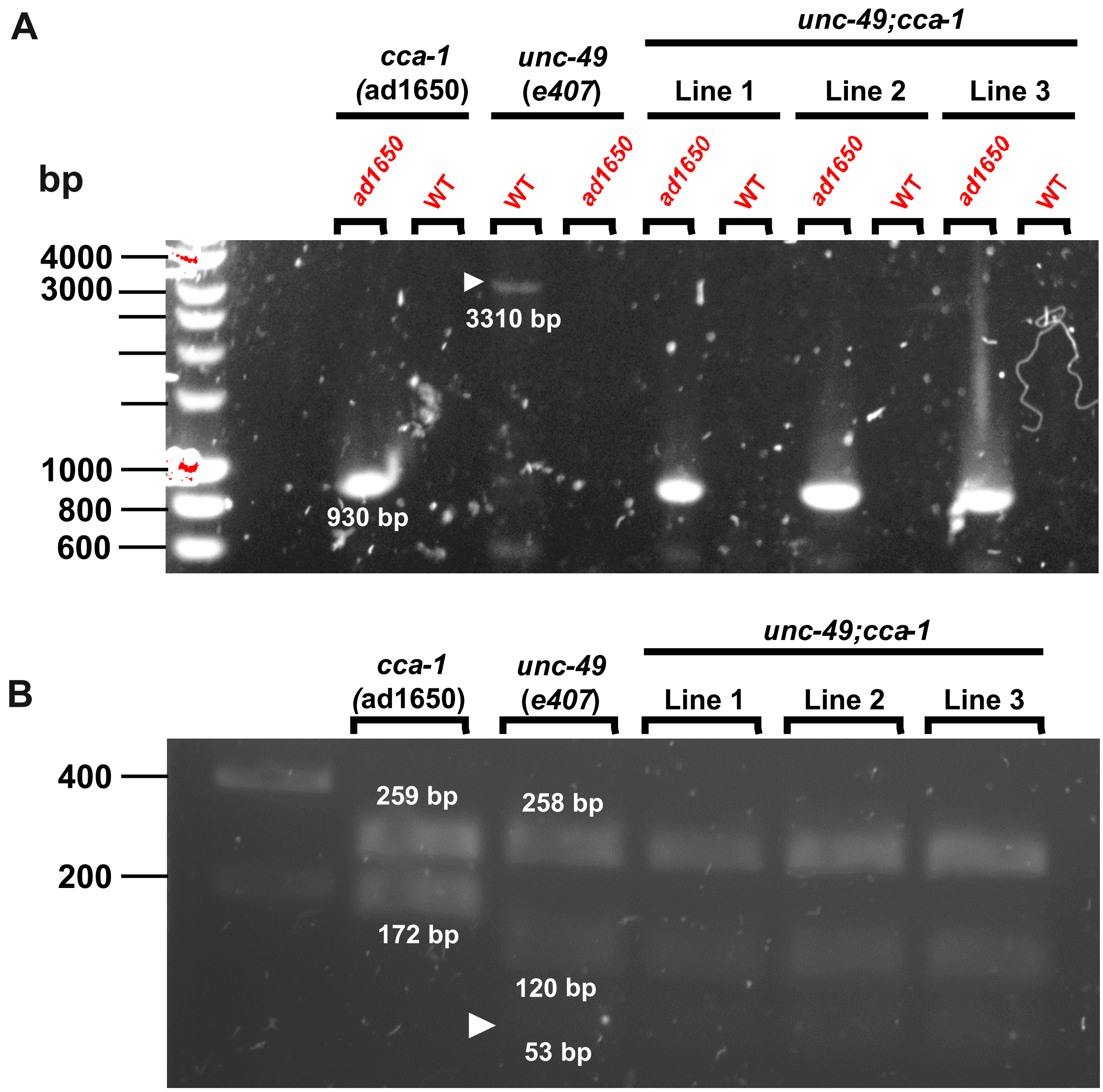

Figure S1: Agarose gels showing verified genotypes of unc-49;cca-1 lines.

Parental mutant strains *cca-1 (ad1650)* and *unc-49 (e407)* were analysed alongside as reference controls for the corresponding mutant and WT alleles. (A) Primers specific for WT and mutant *cca-1* alleles (red) produce PCR products of 3310 and 930 bp respectively. All *unc-49;cca-1* lines were homozygous for the *cca-1 (ad1650)* mutation as only mutant product was detected. (B) Exon 5 of the *unc-49 (e407)* mutant allele contains a C to T point mutation. The exon was amplified with PCR, followed by subsequent differentiation of WT and mutant alleles with *MseI* restriction enzyme digestion, which cuts respective PCR products at one and two T^TAA sites to generate two WT and three mutant cleavage products of the specified sizes; the smallest cleaved fragment from mutant product is very faint and indicated with an arrow head. Three clear cleavage products were present for all lines, confirming *unc-49 (e407)* homozygosity.bp; base pairs.
